# ICAD: A Self-Supervised Autoregressive Approach for Multi-Context Anomaly Detection in Human Mobility Data

**DOI:** 10.1145/3748636.3762774

**Published:** 2025-12-12

**Authors:** Bita Azarijoo, Maria Despoina Siampou, John Krumm, Cyrus Shahabi

**Affiliations:** University of Southern California, Los Angeles, California, USA; University of Southern California, Los Angeles, California, USA; University of Southern California, Los Angeles, California, USA; University of Southern California, Los Angeles, California, USA

**Keywords:** Human Mobility Anomaly Detection, Trajectory Anomaly Detection, Spatiotemporal Data Mining, Interpretable Machine Learning

## Abstract

Abnormal human mobility patterns often signal disruptions, emergencies, or health-related risks, making their detection critical for applications in public safety, urban monitoring, and healthcare. Existing approaches for human mobility anomaly detection typically focus on either identifying visits to unusual places or overall deviations from individual- and population-level norms at the agent-level. However, these methods often (1) overlook fine-grained temporal anomalies, and (2) lack interpretability, as they do not reveal which specific spatiotemporal components of a visit contribute to its anomalous nature. To overcome these limitations, we present ICAD (Interpretable Component-wise Anomaly Detection), a self-supervised autoregressive model that detects both spatial and temporal anomalies by modeling deviations in an individual’s visit-level mobility behavior. ICAD is trained on normal visit sequences using a next-visit prediction objective to learn the distribution of visits under regular conditions. At inference, it computes component-wise anomaly scores for each visit by measuring relative divergence from the learned distribution of normal behavior. Specifically, ICAD proposes a top-k deviation metric for discrete spatial anomalies and introduces a novel relative mode-based scoring function for detecting temporal anomalies in continuous time. Experiments on a large scale synthetic human mobility dataset show that ICAD outperforms prior methods in both visit-level and agent-level anomaly detection. For reproducability purposes, the source code is accessible at https://github.com/USC-InfoLab/ICAD.

## 1 Introduction

Detecting abnormal mobility patterns is crucial for identifying disruptions caused by emergencies, social disturbances, or health-related events. From public safety and disaster response to transportation planning and disease surveillance, many applications depend on fast and accurate detection of these irregularities [1, 6, 17, 24, 28, 31, 35, 36]. These anomalies can occur in both space (e.g., visiting an unusual location), and time (e.g., arriving at a typical location at an unusual hour). As a result, it is crucial to detect and interpret deviations across both spatial and temporal dimensions.

Prior approaches to human mobility anomaly detection have primarily followed two directions. The first line of work defines anomalies as visits to uncommon or rarely observed places, focusing primarily on detecting spatial outliers, such as visits to unexpected points of interest (POIs) or unfamiliar regions. Although temporal attributes (e.g., arrival or departure times) are sometimes included as input features, they are not incorporated into the optimization objective. Consequently, these methods do not explicitly model temporal regularities, which may limit their ability to capture time-dependent anomalies [20, 23]. The second line of work represents each visit as a single embedding that jointly encodes multiple attributes, including location, arrival time, and duration of stay. Anomalies are identified by measuring deviations in these embeddings across entire visit sequences, relative to individual- or population-level behavioral norms. While effective for capturing coarse-grained deviations, these methods treat visits as holistic representations, making it difficult to determine which specific attribute(s) contributed to the anomaly. For instance, when a visit is flagged as anomalous, it remains unclear whether this is due to a visit to an unseen place, the time of arrival/departure from a specific place, or both. This lack of attribute-level interpretability limits the practical utility of such methods in applications that require precise behavioral insights [2, 20, 23, 41].

On the other hand, identifying temporal anomalies presents unique challenges. Unlike spatial anomalies, which often involve discrete locations that can be ranked or categorized based on frequency, temporal attributes are continuous and highly context-sensitive, with their interpretation varying across individuals, cultural norms, and external conditions such as time of day, day of the week, or season. For example, visiting a gym at 10 a.m. may be typical for one user but unusual for another, and staying at a park for two hours may be expected on weekends but not during weekday mornings. To that extent, techniques that detect temporal anomalies typically assign anomaly scores using absolute likelihoods, estimated by neural density or probabilistic models [11]. However, these scores are highly sensitive to the scale and shape of the underlying distributions, making it difficult to define consistent thresholds for anomaly detection across users and regions. As a result, such methods risk misclassifying uncommon but legitimate behaviors as anomalies, while failing to capture subtle deviations from an individual’s established routine. Overcoming these limitations requires temporal scoring methods that incorporate contextual factors, such as personal routines and time-dependent patterns, rather than relying solely on raw likelihood estimates.

Motivated by the aforementioned shortcomings, we present ICAD (Interpretable Component-wise Anomaly Detection), a novel self-supervised autoregressive framework that quantifies *relative*, *multi-context* spatiotemporal deviations from each individual’s typical visit patterns. Specifically, ICAD decomposes each visit into three distinct components: location, arrival time, and departure time. By training on normal mobility patterns via next-visit prediction, ICAD learns the underlying probability distribution of visits under regular conditions. During inference, ICAD leverages this learned distribution to detect anomalous visits in both spatial and temporal dimensions. For discrete locations, ICAD computes anomaly scores based on deviations from the top-k predicted locations. For continuous temporal components, it introduces a novel and consistent scoring method based on the proposed *mode marginal* density derived from Gaussian Mixture Models (GMM), quantifying relative divergence from the most probable regions of the distribution. This component-wise, context-sensitive scoring enables fine-grained interpretability by revealing which specific visit attributes, location, arrival time, or departure time, are anomalous, and why, across different users, places, and temporal settings.

To further clarify our *multi-context* anomaly scoring, we define and categorize spatiotemporal anomalies into three distinct types:

- **Temporal Anomaly**: A deviation in the time of an event relative to an individual’s established routine. For example, an individual visiting a familiar location at an atypical hour.
- **Spatial Anomaly**: A deviation in the location of an event relative to an individual’s established routine. For instance, an individual traveling to an unexpected location during a time typically reserved for commuting to a workplace.
- **Compound Anomaly**: Deviations in both time and space occurring simultaneously. For example, an individual visiting an unfamiliar location at an unusual time of day.

In summary, our contributions are:

- We introduce a novel and *multi-context* anomaly scoring method that jointly fuses spatiotemporal deviations *relative* to normal patterns.
- We propose a novel *mode-margin* scoring technique for continuous temporal variables, which calculates anomaly scores based on the gap between the observed data likelihood and modes of GMM components, thus offering a principled, intuitive measure of relative temporal deviations.
- We demonstrate that the proposed *multi-context* anomaly scoring consistently outperforms alternative scoring techniques from the literature, at both the visit-level and agent-level anomalies.
- Our framework provides interpretability through component-wise scoring, enabling clear identification and interpretation of whether anomalies arise from spatial, temporal, or compound factors.

The remainder of the paper is organized as follows. Section 2 reviews related work on anomaly detection in human mobility data. Section 3 formally defines the problem of *multi-context* anomaly detection. Section 4 presents our proposed ICAD framework, and Section 5 reports our experimental evaluation. Section 6 discusses the limitations of ICAD, including challenges of real-world evaluation, computational complexity, and dataset dependence of temporal signals. Finally, Section 7 concludes the paper.

## 2 Related Work

Anomaly detection in trajectories has long been a prominent research topic in spatiotemporal data mining. The prior methods can be categorized into traditional and deep learning methods.

### 2.1 Traditional Methods

Early methods relied on hand-crafted features to detect anomalies. For instance, TRAOD [18] partitions a trajectory into segments and then combines a distance-based and density-based approach to detect sub-trajectory anomalies. iBOAT [4] introduces an online approach to detect fraudulent taxi routes by monitoring drivers’ greedy routing decisions, while IBAT [39] represents each taxi trajectory as a sequence of symbols and applies an isolation-based method to flag both driving fraud and road network changes. MoNav-TT [40] utilizes graph centrality measurements to measure connection strength between road networks of different parts of New York City and large-scale taxi trips to detect anomalies in taxi trip records.

### 2.2 Deep Learning Methods

Recent deep learning-based methods contributed to the advancement of the field [5]. ATD-RNN [33] proposed a supervised approach which leverages RNNs and a fully connected layer to depict the characteristics of anomalous and normal trajectories. IGMM-GAN [32] uses a CNN-based bidirectional GAN and assumes learned trajectory embeddings follow a multimodal guassian distribution and can be clustered. It then proceeds to calculate the anomaly score on test data by computing the distance between that trajectory and its corresponding cluster centroid. ATROM [12] utilizes variational Bayesian methods to explore behavioral patterns of trajectories under the guidance of probability measure rules, addressing anomaly trajectory recognition in open-world scenarios. Autoencoder-based methods have been widely used for anomaly detection problems [10, 43]. Namely, GM-VSAE [21] adapts an RNN-based VAE model to learn the probability distribution of trajectories in the latent space. Once the VAE model is trained, it leverages the learned generative model to detect anomalies by computing the likelihood of the test trajectories being generated from Gaussian components and improving online computation efficiency. Building upon this, DeepTEA [14] further accounts for the temporal dimension. In terms of anomaly detection in human mobility data, TOD4Traj [41] measures an anomaly detection score by cross-time and cross-population abnormal behaviors. However, it overlooks the arrival and departure time of each visit and only uses day of week as external knowledge in the input. Moreover, it assigns one anomaly score for each sequence of trajectories and fails to predict at the more challenging fine-grained visit level. In addition, LMTAD [23] uses a language model on the next location prediction task to measure the divergence from normal behavior, but it treats duration of stay as a discrete variable by binning it into buckets, an assumption which fails to capture fine-grained continuous nature of temporal anomalies.

Although it is intuitive to leverage next location prediction or contrastive approaches to measure spatiotemporal deviations from normal patterns, they often: (1) fail to detect the fine-grained temporal shift in visit patterns indicating an anomalous behavior, (2) rely on predefined assumptions such as discretizing time or fixed POI categories rather than modeling rich spatiotemporal signals directly in continuous space, (3) lack interpretability, failing to pinpoint which of location or timing caused the anomaly, (4) use absolute anomaly scores rather than measuring relative deviations from the highly probable regions across multiple dimensions. To address these challenges, in Sections 3 and 4, we elaborate on the architecture and design choices of ICAD.

### 3 Problem Formulation

We study the problem of anomaly detection in human mobility by identifying visits that deviate from an agent’s established spatiotemporal patterns. We term this task *multi-context anomaly detection*, as anomalies may arise from irregularities across multiple components of a visit.

Let a *visit* be defined as $v_{i}=\left(r_{i},t_{i}^{a},t_{i}^{d}\right)$, where $r_{i}$ denotes the geographical region cell, $t_{i}^{a}$ the arrival time, and $t_{i}^{d}$ the departure time. For an agent’s visit sequence up to step i-1, $V_{i-1}=\left\langle v_{1},v_{2},\ldots ,v_{i-1}\right\rangle$, we define a probabilistic model $P_{\theta }\left(v_{i}|V_{i-1}\right)$ parameterized by θ that characterizes the distribution of *normal* visits. The parameters are estimated on anomaly-free data by maximum likelihood:

(1) $$\overset{ˆ}{\theta }=arg\;\underset{\theta }{max}\;\prod_{i}P_{\theta }\left(v_{i}|V_{i-1}\right).$$

At inference time, the objective is to quantify the degree to which an observed visit $v_{i}$ diverges from its expected distribution under $P_{\theta }$. We compute component-wise anomaly scores $S_{v_{i}}^{R},$ $S_{v_{i}}^{t^{a}},$ $S_{v_{i}}^{t^{d}}$, corresponding to deviations in the visit’s region, arrival time, and departure time. The final multi-context anomaly score is obtained as a weighted combination:

(2) $$S_{v_{i}}=w_{1}\times S_{v_{i}}^{R}+w_{2}\times S_{v_{i}}^{t^{a}}+w_{3}\times S_{v_{i}}^{t^{d}},$$

where $w_{1},$ $w_{2},$ $w_{3}$ are hyperparameters that balance the contribution of each component. This formulation enables both overall anomaly detection and interpretable attribution of anomalies to specific spatial and temporal contexts.

## 4 Methodology

In this section, we present ICAD’s architecture for detecting multi-context anomalies in human mobility. In our setup, each visit $v_{i}$ is represented by four components: a location $l_{i}$, a region cell $r_{i}$, an arrival time $t_{i}^{a}$, and a departure time $t_{i}^{d}$. To that end, ICAD is trained to estimate the conditional probability distribution of each visit $v_{i}=(l_{i},r_{i},t_{i}^{a},t_{i}^{d})$ given the preceding sequence $V_{i-1}$, thereby capturing joint spatiotemporal regularities in normal behavior. Using the chain rule, we decomposed the distribution as:

(3) $$P\left(v_{i}|V_{i-1}\right)=P\left(r_{i}|V_{i-1}\right)\times P\left({\overset{ˆ}{t}}_{i}^{a}|l_{i},r_{i},V_{i-1}\right)\times P\left({\overset{ˆ}{t}}_{i}^{d}|t_{i}^{a},l_{i},r_{i},V_{i-1}\right)$$

Concretely, the model estimates next visit components as:

- Region cell: $\overset{ˆ}{P}\left(r_{i}|V_{i-1}\right)$
- Arrival time: $\overset{ˆ}{P}\left({\overset{ˆ}{t}}_{i}^{a}|l_{i},r_{i},V_{i-1}\right)$, given the true location $l_{i}$ and region cell $r_{i}$ encodings of $v_{i}$.
- Departure time: $\overset{ˆ}{P}({\overset{ˆ}{t}}_{i}^{d}|t_{i}^{a},l_{i},r_{i},V_{i-1})$, given the true location $l_{i}$, region cell $r_{i}$ and arrival time $t_{i}^{a}$ encodings of $v_{i}$.

During inference, anomaly scores are computed for each component ($S_{v_{i}}^{R},S_{v_{i}}^{t^{a}},S_{v_{i}}^{t^{d}}$) by evaluating deviations from the model’s learned distribution. A weighted fusion then produces the overall anomaly score $S_{v_{i}}=w_{1}\times S_{v_{i}}^{R}+w_{2}\times S_{v_{i}}^{t^{a}}+w_{3}\times S_{v_{i}}^{t^{d}}$, which reflects joint abnormalities across spatial and temporal dimensions.

### 4.1 Visit Sequence Construction

We begin by specifying how each visit is represented in terms of its spatial and temporal components, and then construct the sequence of visits that serves as input to the model.

The location coordinates $l_{i}=\left(x_{i},y_{i}\right)$ of each visit are encoded using Poly2Vec [30]. Poly2Vec models each location as a Dirac delta function and applies a continuous Fourier transform, allowing us to obtain location encodings that preserve the relative distance between the POIs in latent space. The resulting location encoding vector is defined as

(4) $$l_{i}=Poly2Vec\left(x_{i},y_{i}\right),$$

where $l_{i}\in R^{d}$ is obtained by evaluating the Fourier transform on a fixed set of frequency components (u,v).

To ground the next-region prediction and learn structured representations of spatial regions, we further discretize all POI locations into cells using Uber’s H 3 index [13]. Each region cell $c_{i}$ is then mapped to a learnable embedding vector

(5) $$r_{i}=Emb\left(c_{i}\right),$$

where $r_{i}\in R^{d}$ denotes the region representation of visit $v_{i}$.

Similarly, the arrival and departure timestamps of each visit are encoded using Time2Vec [16], which captures periodic and non-periodic temporal patterns. For a timestamp $t_{i}$, the encoding is given by

(6) $$t_{i}=Timen2Vec\left(t_{i}\right),$$

where $t_{i}\in R^{d}$ represents the temporal embedding of visit $v_{i}$.

Finally, we construct the visit sequence representation H by concatenating the encodings of all contextual components:

(7) $$H=\left[l;\;r;\;t^{a};\;t^{d}\right],$$

where $l,r,t^{a},t^{d}\in R^{m\times d}$ denote the encoded locations, region cells, arrival times, and departure times for each visit in the sequence, respectively. Here, m is the sequence length and d is the embedding dimension, yielding

$$H\in R^{m\times 4d}$$

The operator “;” denotes concatenation along the feature dimension.

### 4.2 Visit Sequence Encoding

Next, to capture joint spatiotemporal dependencies, we pass the full visit sequence representation H through a causal transformer encoder. The causal attention mechanism restricts each step to attend only to preceding visits, thereby enforcing autoregressiveness while modeling cross-dimensional dependencies in location, region, and time.

To preserve the original sequential order of visits, we add positional encodings (PE) to H prior to passing it into the transformer encoder [37]. The resulting representation given by:

(8) E=CausalTransformerEncoder(H+PE(H)),

where $E\in R^{m\times 4d}$ denotes contextual visit embeddings.

### 4.3 Visit Component Prediction

As mentioned, ICAD is optimized to maximize the likelihood of the next visit given its history. To that extent, we decompose this prediction into its constituent components; region, arrival time, and departure time, and describe each one in the following sections.

#### 4.3.1 Region Cell Prediction.

We formulate next-region prediction of a next visit, as a multi-class classification task over the set of all regions R. Specifically, given the encoded visit sequence E, an additional causal transformer encoder is applied, followed by a linear projection and a softmax layer to obtain the probability distribution:

(9) $$\overset{ˆ}{P}\left(r_{i}|V_{i-1}\right)=Softmax(Linear(CausalTransformerEncoder(E))),$$

where the softmax output lies in $R^{m\times |R|}$, with m denoting the visit sequence length and |R| being the number of regions.

#### 4.3.2 Arrival Time-of-day Prediction.

We predict the arrival time-of-day of the next visit $v_{i}$ in the sequence by modeling the travel time from the previous visit. Specifically, we define travel time as the difference between the arrival time (in hours) of visit $v_{i}$ and the departure time (in hours) of the previous visit $v_{i-1}:\Delta t_{i}^{T}=t_{i}^{a}-t_{i-1}^{d}$, and train the model to estimate the conditional distribution of $\Delta t_{i}^{T}$. Note that alternatively, the model could be trained to directly predict the absolute arrival time of day for the next visit. However, in our experiments we found that modeling travel time yielded more stable and accurate results. We further discuss this choice in Section 5.6.2.

To parameterize the distribution of $\Delta t_{i}^{T}$, we employ Gaussian Mixture Models (GMM) which has been shown to effectively capture continuous temporal variables [15]. The GMM parameters are predicted through a feedforward (FF) layer applied to the output of the causal cross-attention (${Attn}_{\text{cross}}$). Specifically, we define the query (Q) as

(10) $$p_{i}=\left[1_{i};\;r_{i}\right],$$

where $l_{i}$ and $r_{i}$ denote the true location and region encodings of visit $v_{i}$, respectively. Furthermore, the visit sequence encoding $E_{i-1}$ of the sequence of visits prior to $v_{i}$ acts as both the keys (K) and values (V). The cross-attention output is then mapped to the GMM parameters:

(11) $$\overset{ˆ}{P}\left(\Delta t_{i}^{T}|r_{i},V_{i-1}\right)=GMM\left(FF\left({Attn}_{\text{cross}}\left(p_{i},E_{i-1},E_{i-1}\right)\right)\right)$$

Each predicted parameter of the GMM output lies in $R^{m\times k}$, where k denotes the number of Gaussian components. This design allows the model to generate GMM parameters that are contextually grounded in the spatial and temporal history of a user’s visits, thus enabling accurate modeling of the arrival-time distribution.

#### 4.3.3 Departure Time-of-day Prediction.

Similar to arrival time prediction, we model the departure time-of-day ${\overset{ˆ}{t}}_{i}^{d}$ of the next visit $v_{i}$ by estimating the parameters of a separate Gaussian Mixture Model (GMM). This estimation is conditioned on the true location $l_{i}$, region cell $r_{i}$, the arrival time $t_{i}^{a}$ encoding vectors, as well as the the encoded sequence of prior visits $E_{i-1}$ of preceding visits. To this end, we first construct a context vector

(12) $$d_{i}=\left[l_{i};\;r_{i};\;t_{i}^{a}\right],$$

Then, the model predicts the distribution of the departure time through a causal cross-attention module:

(13) $$\overset{ˆ}{P}\left({\overset{ˆ}{t}}_{i}^{d}|l_{i},r_{i},t_{i}^{a},V_{i-1}\right)=GMM\left(FF\left({Attn}_{\text{cross}}\left(d_{i},E_{i-1},E_{i-1}\right)\right)\right)$$

As in the arrival-time head, the predicted GMM parameters lie in $R^{m\times k}$, where m is the sequence length and k is the number of Gaussian components. Note that in this formulation we directly predict departure time rather than duration of stay, which could be defined as the difference between the departure time (in hours) and the arrival time (in hours) of visit $v_{i}:\Delta t_{i}^{D}=t_{i}^{d}-t_{i}^{a}$. A comparison of different temporal modeling choices is reported in Section 5.6.2.

### 4.4 Training Objective

During training, ICAD is exposed only to regular (i.e., non-anomalous) visit patterns. ICAD is trained to predict the components of the next visit by maximizing their likelihood under the learned data distribution. This is equivalent to minimizing the negative log-likelihood (NLL) of the joint distribution over the predicted region cell, arrival time, and departure time of each visit. To that extent, the training objective is defined as:

(14) $$ℒ\;=-\frac{1}{N}\sum_{i=1}^{N}\;log\;\overset{ˆ}{P}\left(r_{i},t_{i}^{a},t_{i}^{d}|V_{i-1}\right)\;\;=-\frac{1}{N}\sum_{i=1}^{N}\;log\left[\overset{ˆ}{P}\left(r_{i}|V_{i-1}\right)\overset{ˆ}{P}({\overset{ˆ}{t}}_{i}^{a}|r_{i},V_{i-1})\overset{ˆ}{P}({\overset{ˆ}{t}}_{i}^{d}|r_{i},t_{i}^{a}V_{i-1})\right]\;\;=-\frac{1}{N}\sum_{i=1}^{N}\;\left[log\overset{ˆ}{P}\left(r_{i}|V_{i-1}\right)+log\;\overset{ˆ}{P}({\overset{ˆ}{t}}_{i}^{a}|r_{i},V_{i-1})+log\;\overset{ˆ}{P}({\overset{ˆ}{t}}_{i}^{d}|r_{i},t_{i}^{a},V_{i-1})\right]$$

where N is total number of visits at training time.

### 4.5 Multi-Context Anomaly Score Calculation

**Figure 2:**
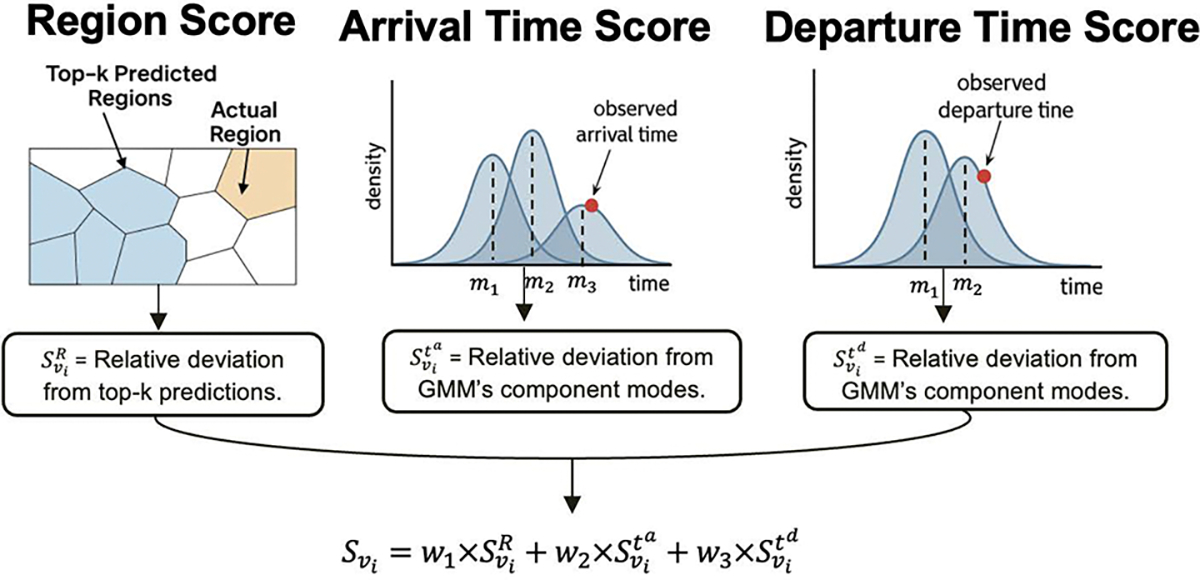
Overview of ICAD’s anomaly score calculation. Anomaly score of a visit is a weighted combination of each component’s score.

At inference time, unlike training, agents may deviate from their usual visit patterns, either by traveling to unexpected locations, or showing abnormal patterns in arrival or departure times, or both. To capture these multi-context deviations, we compute component-wise anomaly scores. Specifically, we categorize visit components into two types: components in discrete space (e.g., region cells) and continuous space (e.g., arrival and departure times), and design separate scoring strategies. For all components, we compute scores using log-likelihoods to ensure numerical stability and consistency across prediction ranges. Figure 2 shows an overview of the anomaly score calculation.

#### 4.5.1 Discrete Space.

To quantify spatial anomalies for predicted regions compared to the actual visited ones, we utilize the probability mass function (PMF) calculated over all regions in Section 4.3.1. Knowing that regions are mutually exclusive since an agent can not be in multiple regions in one visit, we have:

(15) $$\sum_{i=1}^{\left|R\right|}P\left(r_{i}|V_{i-1}\right)=1.$$

Therefore, we compute the regions anomaly score as the relative log-likelihood of the actual visited regions compared to the model’s top predicted regions. To ensure flexibility across diverse regular patterns, we define a reference set consisting of the top-k most probable predicted regions denoted as ${𝒯}_{k}=TopK(\overset{ˆ}{P}\left(.|V_{i-1}\right),k)$. If the true region $r_{i}$ falls within ${𝒯}_{k}$, we assign an overall score of zero; otherwise, we measure the average deviation from the top-k predicted regions using its relative log-likelihood. Specifically, we compute:

(16) $$S_{v_{i}}^{R}=\left\{\begin{array}{ll} 0, & \text{if}\;r_{i}\in {𝒯}_{k,}\\ \frac{1}{k}\sum_{r_{j}\in {𝒯}_{k}}\;log\overset{ˆ}{P}\left(r_{j}|V_{i-1}\right)-log\overset{ˆ}{P}\left(r_{i}|V_{i-1}\right), & \text{otherwise.} \end{array}\right.$$

where $S_{v_{i}}^{R}$ refers to the region anomaly score for the i’th visit. Note that the probability of each of the top-k predicted region is always greater than or equal to the probability of the actual visited region. As a result, the anomaly score is guaranteed to be non-negative.

#### 4.5.2 Continuous Space.

Unlike discrete space, where probabilities are defined over individual outcomes using the PMF, continuous space relies on a probability density function (PDF), which assigns probability over intervals. Since PDFs represent densities rather than actual probabilities, their values can exceed 1. Consequently, directly using log-likelihoods of predicted densities can lead to interpretational issues: high-density values may produce likelihoods greater than one, while low-density values yield negative values. This inconsistency complicates the integration of continuous anomaly scores with discrete ones, such as region-based scores, which are always non-negative.

To address this, we compute anomaly scores for continuous variables relative to the log-likelihood of the marginal density evaluated at the modes of the GMM components. In a Gaussian Mixture Model (GMM), each component is a Gaussian distribution whose mode $m_{k}$ equals its mean $\mu_{k}$. Accordingly, we calculate anomaly score as:

(17) $$S_{v_{i}}^{t}=log\left(\Sigma_{k}\pi_{k}f_{k}\left(m_{k}\right)\right)-log\left(\Sigma_{k}\pi_{k}f_{k}(t)\right)$$

Here, $\pi_{k}$ denotes the prior mixture probability of the k’th component, and $f_{k}(t)$ refers to the PDF of the k’th GMM component at time t. Notably, for any value of t, the term $\Sigma_{k}\pi_{k}f_{k}\left(m_{k}\right)$ is an upper bound of the GMM output. The complete proof is in appendix A.1. This relative scoring offers several key advantages:

1. It captures instance-specific deviations relative to the high-density regions of the GMM. For example, consider a visit pattern with strong peaks at 8:00 AM and 9:00 PM, and a smaller peak around 1:00 PM, perhaps reflecting a recent behavioral shift, such as an occasional midday stop at a gas station. The relative scoring mechanism effectively accounts for such subtle variations by comparing observed behavior to the model’s highest-density patterns (i.e. normal behaviors).
2. By relying on multiple Gaussian components, the method can capture recurring visit times that occur at different parts of the day. This allows the anomaly score to reflect both dominant regularities (e.g., daily commute times) and less frequent but still systematic behaviors (e.g., occasional bi-weekly midday stops at a gas station), rather than being biased toward a single peak in the distribution.
3. It guarantees that anomaly scores remain non-negative, enabling consistent integration with discrete anomaly scores.

Other approaches for reconciling probability mass and probability density include (1) discretizing the density function and (2) integrating the density function over a small interval around the assigned value. Both require the manual choice of a resolution parameter (e.g., bin width or interval size). In contrast, our relative scoring does not introduce any additional hyperparameters: the reference values are automatically determined by the fitted GMM components. This makes the method especially suitable for GMMs, where component modes are well-defined.

#### 4.5.3 Multi-Context Anomaly Scoring.

After obtaining the region anomaly score $S_{v_{i}}^{R}$ following the methodology detailed in Section 4.5.1, and arrival time and departure time anomaly scores following Section 4.5.2, we compute the total anomaly score as a weighted combination of each each of its components:

(18) $$S_{v_{i}}=w_{1}\times S_{v_{i}}^{R}+w_{2}\times S_{v_{i}}^{t^{a}}+w_{2}\times S_{v_{i}}^{t^{d}}$$

where $w_{1},$ $w_{2}$, and $w_{3}$ are hyperparameters balancing among multi-context anomaly scores. This multi-context scoring framework also provides interpretability by indicating whether an anomaly stems from spatial, temporal, or combined deviations when any component significantly diverges from its normal pattern.

## 5 Experiments

This section analyzes ICAD’s performance compared to the state-of-the-art baselines. It discusses ICAD’s superior results based on modeling relevant spatiotemporal signals and our novel approach in multi-context relative anomaly scoring.

### 5.1 Dataset

Existing benchmarks for human mobility anomaly detection are often limited by their smaller scale or by simplified, predefined behavioral assumptions that are uniformly applied to agents in the simulation, which limits their extensibility for visit-level anomaly detection [44]. To provide a more comprehensive evaluation, we conduct our experiments on the NUMOSIM dataset [34], a large-scale synthetic benchmark that models diverse human mobility behaviors across both spatial and temporal dimensions. Table 2 shows the statistics of this dataset.

NUMOSIM simulates visits of 200,000 agents in Los Angeles County over eight weeks, starting in January 2024 [34]. The first four weeks are used for training and contain only normal visit patterns, while the remaining four weeks are used for evaluation and contains two major anomalous behaviors: (1) non-recurring disruptions, representing occasional spatial deviations from regular visit patterns, and (2) recurring deviations, defined as consistent temporal shifts in arrival and departure times at routine places. For example, visiting a distant doctor’s office in the middle of a working day is a non-recurring disruption in one’s regular visit patterns. Subsequently, it causes an early departure from work and a delayed return back to work, both of which represent temporal anomalies. For the rest of the paper, we refer to recurring anomalies as *temporal anomalies* and non-recurring anomalies as *spatial anomalies*.

NUMOSIM is intended for visit-level anomaly detection. For agent-level analysis, we consider an agent anomalous if they have at least one anomalous visit. The agent-level anomaly score is then defined as the maximum anomaly score observed across all visits in an agent’s entire visit sequence.

### 5.2 Baselines

We compare ICAD to state-of-the art unsupervised and self-supervised anomaly detection methods in literature.

- **STOD** [7] is a GRU-based model which leverages entropy of the probability distribution over bus route IDs to determine anomalies in bus trajectories.
- **RioBusData** [3] is an open source tool that uses a CNN to detect bus routes anomalies.
- **GM-VSAE** [21] introduces a Gaussian Mixture Variational Sequence Auto Encoder (GM-VSAE), which uses a Gaussian Mixture to learn normal behavior as a prior for a generative network responsible of detecting anomalies in taxi data.
- **DeepBayesics** [11] encodes agent IDs along with arrival time, POI activity types, and duration of stay to predict next visit components. It then measures how far the parameters of the actual next visit deviate from the predicted visit under a non-anomalous setting.
- **LMTAD** [23] employs an autoregressive causal-attention model over discretized trajectory tokens, trained to predict the next location given historical context. Anomalies are detected as low-probability tokens using perplexity at the trajectory level and surprisal at the visit level.
- **TOD4Traj** [41] employs both feature-level and trajectory-level contrastive learning objectives to fuse spatial, temporal, and semantic information and capture repetitive mobility patterns within and across agents. It further defines two types of anomaly scores: one measuring an individual’s deviation from its own regular visit patterns, and another capturing deviation from normal behavior at the population level.

### 5.3 Evaluation Metrics

To evaluate component-wise anomaly detection performance, we use Average Precision (AP) and Area Under Receiver Operator Characteristic (AUROC). AP is computed from the weighted average of precisions across different levels of recall, and is suitable for measuring extremely rare anomalies in skewed datasets [9, 21, 42], while AUROC measures a model’s ability to assign higher scores to true anomalies compared to normal points regardless of class imbalance. Both of these metrics are widely used for anomaly detection [41].

### 5.4 Experimental Setup

We report the best results for all baselines using their official implementations and recommended hyperparameters. To adapt LMTAD for visit-level evaluation, we report the negative log-likelihood of the predicted location, as specified in its objective function. GM-VSAE and TOD4Traj assign one score to the entire visit sequence at the agent level. Consequently, we cannot report their performance at visit-level.

#### 5.4.1 Hardware Configurations.

All exepriments were conducted on a machine equipped with an an NVIDIA A100 80GB GPU and an AMD EPYC 7V13 64-core CPU. The software environment was configured with Python 3.10.13, PyTorch 2.5.1 and CUDA 12.2.

#### 5.4.2 Hyperparameter Details.

The summary of hyperparameters used in this work is as follows:

- **Poly2Vec** [30]: We followed the original paper’s configuration by setting the minimum frequency to 0.1, maximum frequency to 1.0, and total number of sampled frequencies to 210.
- **Training**: During training, we used Adafactor [26] as the optimizer, with learning rate 0.01, transformer dropout rate 0.1, and batch size 512. For transformer-based modules, we used 4 layers with 2 attention heads for encoding and decoding. We trained the model for 300 epochs and chose the one with highest AP and AUROC to report the results.
- **Embedding Dimensions**: We set the embedding size d of a visit’s component to 32. Subsequently, the dimension of visit sequence encoding E was 128. The size of the linear feed forward layer was also set to 32.
- **Gaussian Mixture Models**: We model the distributions of arrival and departure times using a Gaussian Mixture Model (GMM) with k=3 components. For each component, the model predicts the mixture weight, mean, and variance.
- **Anomaly Scoring**: The value of k for measuring discrete region divergence was set to 3, chosen from 1, 2, . . . , 10 based on the configuration that yielded the highest performance. For the weighted fusion step, we set $w_{1}=0.3,$ $w_{2}=0.45$, and $w_{3}=0.25$, selected via grid search over 0.05, 0.1, 0.15, . . . , 1 to maximize the separation between normal and anomalous visits.

### 5.5 Anomaly Detection Results

In this section, we evaluate ICAD against state-of-the-art baselines. Table 3 summarizes results on the NUMOSIM dataset under both visit-level and agent-level evaluation. Across both settings, ICAD consistently outperforms all baselines. The closest competitors are DeepBayesics, LMTAD, and TOD4Traj, as they share methodological similarities with our approach: LMTAD is trained for next-location prediction, while DeepBayesics incorporates multiple attributes (duration, POI type, and arrival time), and TOD4Traj aligns spatial-temporal features with semantic information through contrastive learning. Despite these similarities, ICAD achieves substantially stronger performance due to several important design differences. These can be grouped into three categories:

1. **Anomaly scoring**. LMTAD relies on survival rate for next-location prediction, which reduces anomaly detection to the probability of rare location transitions and ignores temporal irregularities. DeepBayesics instead computes the complement of the joint likelihood of stay-point components, including POI type, arrival time, and duration. This approach captures only extreme deviations and suffers from numerical instability due to probability multiplication. TOD4Traj quantifies anomalies by comparing trajectory-level embeddings against historical patterns and population-wide trends, which is effective for broad trajectory outliers but cannot localize anomalies at the visit level. In contrast, ICAD jointly predicts spatial and temporal signals and applies relative scoring in logarithmic space, ensuring numerical stability while enabling the detection of both subtle visit-level irregularities and larger trajectory-level deviations.
2. **Spatiotemporal modeling**. Although LMTAD, DeepBayesics, and TOD4Traj share some modeling intuitions with ICAD, each has key limitations. LMTAD focuses exclusively on next-location prediction and ignores temporal signals, preventing it from capturing anomalies in arrival or departure times. DeepBayesics compresses an entire sequence into a global agent embedding via a transformer autoencoder, discarding sequential dependencies that are fundamental to human mobility. Moreover, its pretraining objective is based on MSE reconstruction, which tends to memorize trajectories rather than learning predictive dynamics useful for anomaly detection. TOD4Traj aligns spatial-temporal features with semantic POI categories through contrastive learning and aggregates visits into trajectory-level embeddings, which limits its ability to detect fine-grained anomalies at the visit level. In contrast, ICAD models visits autoregressively, conditioning each prediction on all preceding visits, and parameterizes arrival and departure times using Gaussian Mixture Models (GMMs). This design preserves sequential order, explicitly models continuous temporal distributions, and enables the detection of both subtle visit-level deviations and broader agent-level anomalies.
3. **Feature encoding and representation**. TOD4Traj embeds location and time as categorical tokens using standard embedding layers, while LMTAD discretizes stay duration into buckets and applies a similar embedding scheme for location and time. DeepBayesics uses one-hot encoding for POI type and min–max normalization for arrival time and duration, feeding these features into a transformer autoencoder that compresses an entire sequence into a global agent embedding. These representations are either coarse (TOD4Traj, LMTAD) or overly reductive (DeepBayesics), limiting their ability to capture fine-grained spatial proximity or temporal periodicity. In contrast, ICAD employs Poly2Vec and Time2Vec to encode spatial and temporal signals in continuous vector spaces, where Poly2Vec preserves spatial distance between region cells and Time2Vec captures both periodicity and temporal shifts. This richer encoding enables more expressive modeling of when and where visits occur, forming the basis for accurate next-visit prediction and fine-grained anomaly detection.

### 5.6 Ablation Study

#### 5.6.1 Anomaly Score Components.

This section examines the contribution of each anomaly score component to ICAD’s performance. We evaluate four variants: (1) **W/O region**, which removes $S_{v_{i}}^{R}$, the average deviation from top-k predictions for location; (2) **W/O arrival**, which removes $S_{v_{i}}^{t^{a}}$, the deviation from expected arrival times; (3) **W/O departure**, which removes $S_{v_{i}}^{t^{d}}$, the deviation from expected departure times; and (4) **W/O weighted** fusion, which replaces the weighted combination of component scores described in Section 4.5.3 with an unweighted sum. Table 4 summarizes the results. We observe that removing arrival-time scoring leads to the largest performance drop across NUMOSIM, highlighting its critical role in anomaly detection: individuals typically maintain consistent arrival-time patterns when visiting regular locations, making deviations highly indicative of abnormal behavior. Moreover, eliminating weighted fusion yields the second-largest degradation in three out of four evaluation settings, underscoring the importance of balancing spatial and temporal scores rather than treating them equally. These results demonstrate that combining region, arrival, and departure signals in a weighted manner is essential for maximizing detection accuracy, with arrival-time deviations contributing most strongly to the overall performance.

#### 5.6.2 Effect of Temporal Signal Modeling.

We further investigate the impact of different temporal signal choices on anomaly detection performance. Table 5 compares variants of ICAD that use either absolute timestamps (arrival and departure times) or proxy signals (travel time and duration). The results show that absolute arrival and departure times yield more informative signals than duration alone, which provides weaker performance. Among all variants, combining travel time with departure time achieves the highest accuracy at both the visit and agent level, indicating that relative measures of mobility (travel time) paired with absolute departure signals capture user regularities most effectively. More broadly, these results indicate that the most informative temporal signal can vary depending on dataset characteristics. For example, datasets dominated by work–commute routines, where people consistently arrive at fixed times (e.g., office arrivals around 9 AM), may benefit more from absolute arrival-time modeling. In contrast, datasets with more flexible or heterogeneous travel behavior, such as those covering leisure or irregular activities, may favor travel-time–based formulations that capture variability in movement between visits. These findings highlight the importance of carefully selecting temporal signals, as different formulations can significantly influence anomaly detection outcomes.

### 5.7 Relative Anomaly Scoring

This section examines the advantages of our novel component-wise *relative* anomaly scoring compared to conventional anomaly scoring methods. We evaluate four approaches:

1. **Joint Likelihood Divergence (JLD)**. Adopted in DeepBayesics, this method computes the complement of the joint likelihood of visit components:
$$S_{v_{i}}\;=1-\overset{ˆ}{P}(r_{i},{\overset{ˆ}{t}}_{i}^{a},{\overset{ˆ}{t}}_{i}^{d}|V_{i-1})\;\;=1-\overset{ˆ}{P}(r_{i}|V_{i-1})\overset{ˆ}{P}({\overset{ˆ}{t}}_{i}^{a}|r_{i},V_{i-1})\overset{ˆ}{P}({\overset{ˆ}{t}}_{i}^{d}|r_{i},{\overset{ˆ}{t}}_{i}^{a},V_{i-1})$$
2. **Negative Log-Likelihood (NLL).** This approach assigns the negative log of a visit’s likelihood under ICAD’s predictive distribution for the next visit.
3. **Bilateral Cumulative Distribution Function (Bi-CDF).** This method quantifies how extreme an observed value is under the learned probability distribution [8, 19, 27]. Since anomalies are expected in the tails, for continuous components (arrival and departure times) we compute both the CDF F(x) and its complement 1-F(x), taking the smaller of the two tail probabilities as the anomaly score. The region anomaly score is computed separately according to Section 4.5.1. Full details are provided in Appendix A.2.
4. **Relative Scoring (Ours).** ICAD introduces a multi-context component-wise scoring scheme that measures the deviation of observed visit components relative to the model’s highest-density regions (see Section 4.5). This approach ensures non-negative, numerically stable scores and allows fine-grained attribution of anomalies across spatial and temporal dimensions.

As shown in Table 6, our proposed relative anomaly scoring consistently outperforms all probability-based alternatives. JLD performs the worst because multiplying probability terms across components introduces numerical instability and biases the score toward detecting only extreme deviations. At the visit level, we observe a substantial AP gap between relative scoring and Bi-CDF. This is due to the multimodal structure of the GMM for temporal components: between two high-density peaks, the GMM forms a low-density valley, and observations in such valleys yield moderate values for both F(x) and 1-F(x), causing Bi-CDF to underestimate their anomalousness. In contrast, relative scoring compares observations against the marginal density of all GMM component modes, ensuring that valley points are still assigned appropriately high anomaly scores. Compared to NLL, relative scoring improves AP by 3.0% and AUROC by 2.0%, which is a meaningful gain in the context of extremely rare anomalies. At the agent level, the differences across methods narrow because aggregating scores across many visits smooths out local variations and highlights only the strongest outliers. Nevertheless, our relative scoring still yields the highest results, especially in AP, underscoring its advantage in ranking rare anomalies.

### 5.8 Component-wise Interpretability of Anomaly Scores

**Figure 3:**
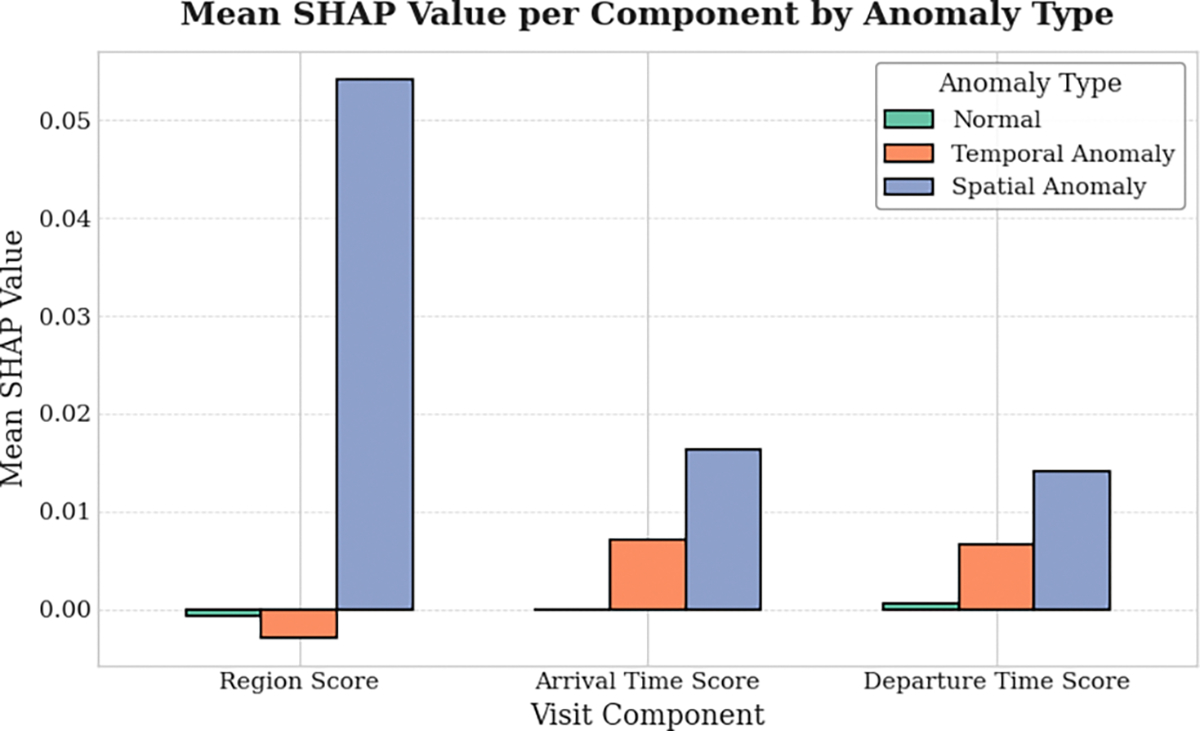
Component-wise SHAP analysis of anomaly scores across visit types (normal, spatial anomalies, temporal anomalies) in NUMOSIM dataset.

A key advantage of ICAD over prior models is that it outputs anomaly scores for each visit component—region, arrival time, and departure time, allowing us to identify which factor makes a visit anomalous. To demonstrate this interpretability, we compute SHAP (SHapley Additive exPlanations) values [22] for normalized component scores across three visit types: normal, spatial anomalies, and temporal anomalies. SHAP values quantify each component’s marginal contribution to the gap between a visit’s anomaly score and the model average, following Shapley-value principles [25]. Positive values push predictions toward “anomaly”, negative values toward “normal”, and their magnitude reflects influence strength. Figure 3 shows mean SHAP values per component. For spatial anomalies, region scores dominate, while arrival and departure times also contribute positively, indicating timing is atypical when visiting unusual locations. For temporal anomalies, the region score contributes negatively (reinforcing normalcy at regular locations), whereas arrival and departure scores are strongly positive, confirming that temporal deviations drive these anomalies. For normal visits, all components have near-zero SHAP values, showing no undue bias toward anomaly labeling. In summary, ICAD not only achieves accurate anomaly detection but also provides interpretable, component-wise explanations: region deviations explain spatial anomalies, timing deviations explain temporal anomalies, and normal visits remain unaffected by any component score.

## 6 Discussion

In this section, we discuss the limitations of ICAD that highlight both the challenges of anomaly detection in human mobility data and opportunities for future research.

- **Evaluation on synthetic data.** Our experiments are restricted to synthetic benchmarks such as NUMOSIM because real-world anomaly evaluation is inherently difficult. First, large-scale human mobility datasets are rarely publicly available due to privacy constraints, which limits access to the raw data needed for anomaly detection research. Second, even when trajectory data are available, they have to be attributed to POIs or regions to provide semantic context, a process that is inherently noisy and ambiguous. To that extent, potential misattributions can generate spurious anomalies that are artifacts of preprocessing rather than genuine behavioral deviations [29]. Third, existing real-world mobility datasets do not contain ground-truth anomaly labels, requiring annotation by human experts, which is a costly and labor-intensive process at scale. Prior works have attempted to circumvent this by injecting synthetic anomalies into real-world trajectories, for example by swapping trajectories between agents or shifting parts of trajectories according to predefined spatiotemporal rules [23, 38, 41]. However, these approaches implicitly assume that the original real-world trajectories are entirely normal, overlooking the fact that genuine anomalies may already be present in the dataset before any perturbations are applied. As a result, injected anomalies may coexist with natural ones, confounding evaluation. Taken together, these challenges make large-scale, reliable anomaly evaluation on real mobility data highly challenging at present, motivating the use of synthetic datasets for controlled experimentation.
- **Computational complexity.** Similar to previous approaches, ICAD employs a transformer backbone to capture spatiotemporal dependencies, which scales quadratically with sequence length. Although human mobility trajectories typically contain relatively few daily visits (e.g., fewer than 15), making the computation manageable in our setting, scalability could become an issue in domains with longer sequences or larger-scale applications.
- **Dataset dependence of temporal signals.** Our ablation studies show that anomaly detection performance is sensitive to the choice of temporal signals, such as arrival time, departure time, duration, or travel time. The most informative representation depends on dataset characteristics. For instance, datasets dominated by strict commuting routines may benefit from absolute arrival times, while more irregular activity datasets may favor relative signals like travel time. This suggests that no single temporal formulation is universally optimal, and adaptive or hybrid approaches may be needed in practice.

Overall, ICAD demonstrates consistently strong results compared to state-of-the-art baselines, and the identified challenges represent important opportunities for future research in human mobility anomaly detection.

## 7 Conclusion

In this work, we introduced ICAD, a novel self-supervised framework for multi-context anomaly detection in human mobility data. ICAD captures deviations across spatial and temporal dimensions by modeling the conditional distributions of individual visit components and computing interpretable, component-wise anomaly scores. By incorporating relative scoring strategies such as top-k deviation for discrete regions and a novel temporal score based on divergence from mode-marginal density of continuous time estimators, ICAD enables principled, fine-grained detection of abnormal visit behaviors within complex mobility patterns. Extensive experiments demonstrated that ICAD outperforms existing methods in anomaly detection while offering deeper insights into the nature of spatiotemporal anomalies.

## Figures and Tables

**Figure 1:**
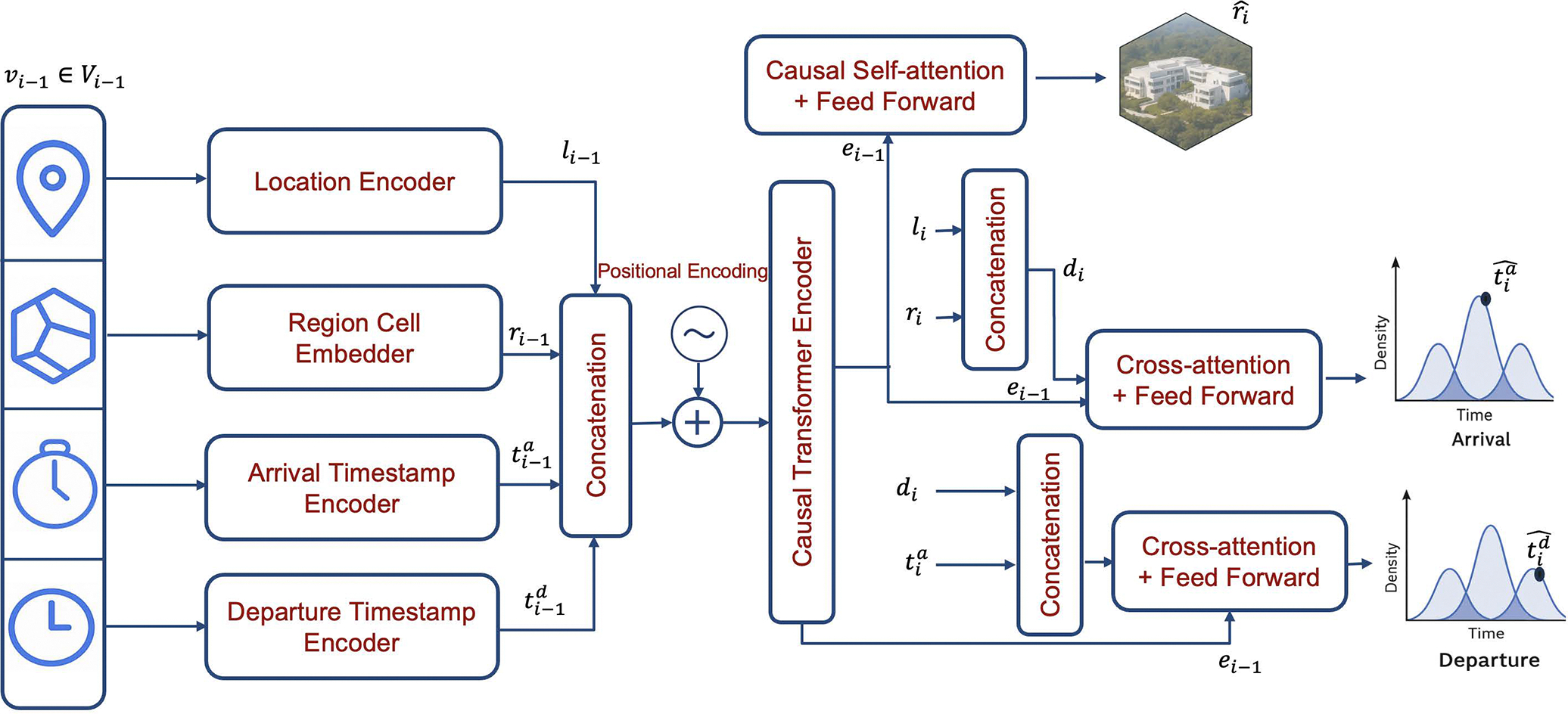
Training pipeline of ICAD for predicting the *i*-th visit $v_{i}$ given the preceding visits $V_{i-1}$.

**Table 1:** Notations used in ICAD.

Symbol	Description

*v_i_*	Visit *i* in a sequence of visits.
*V_m_*	Sequence of visits of length *m*.
*l_i_*	Location coordinates of the *i*’th visit.
l_i_	Location encoded vector of the *i*’th visit.
*r_i_*	Region cell of the *i*’th visit.
r_i_	Region cell encoded vector of the *i*’th visit.
$t_{i}^{a}$	Arrival timestamp of the *i*’th visit.
$t_{i}^{a}$	Encoded arrival time vector of the *i*’th visit.
${\overset{ˆ}{t}}_{i}^{a}$	Predicted arrival time-of-day of the *i*’th visit.
$t_{i}^{d}$	Departure timestamp of the *i*’th visit.
${\overset{ˆ}{t}}_{i}^{d}$	Predicted departure time-of-day of the *i*’th visit.
*H*	Contextual representation of visit sequence *V*.
*E*	Encoding representation of visit sequence *V*.
$\Delta t_{i}^{T}$	Travel time of the *i*’th visit.
*R*	set of all possible region cells.
$S_{v_{i}}^{R}$	Region anomaly score of the *i*’th visit.
$S_{v_{i}}^{t^{a}}$	Arrival anomaly score of the *i*’th visit.
$S_{v_{i}}^{t^{d}}$	Departure anomaly score of the *i*’th visit.

**Table 2:** Statistical details of the NUMOSIM dataset.

Dataset	Granularity	Anomaly Type(s)	Period	# Agents	# Anomalies
NUMOSIM	Visit-level	Spatial & Temporal	28 + 28 d	200,000	3,468
	Agent-level				381

**Table 3:** Performance comparison with the state-of-the-art on NUMOSIM dataset for visit-level and agent-level anomalies. Models are evaluated based on AP and AUROC metrics. Best (in bold) and second best performing models are highlighted.

Model	Visit-level		Agent-level	
	
	AP	AUROC	AP	AUROC

STOD	0.00024	0.5940	0.00182	0.5180
RioBusData	0.00019	0.5050	0.00164	0.5010
GM-VSAE	—	—	0.00192	0.5070
DeepBayesics	0.0042	0.6598	0.0121	0.7258
LMTAD	0.0196	0.5472	0.0020	0.5020
TOD4Traj	—	—	0.0022	0.5256
ICAD	**0.1610**	**0.8332**	**0.2561**	**0.9010**

**Table 4:** Ablation study results on NUMOSIM dataset for visit and agent-level anomalies. Best (in bold) and second best performing models are highlighted.

	Visit-level		Agent-level	
		
	AP	AUROC	AP	AUROC

W/O Region	0.1419	**0.8350**	0.2400	0.8413
W/O Arrival	0.0111	0.6794	0.0287	0.6443
W/O Departure	0.1506	0.7415	0.2265	0.7861
W/O Weighted Fusion	0.1448	0.8258	0.2521	0.8847
ICAD	**0.1610**	0.8332	**0.2561**	**0.9010**

**Table 5:** Ablation on temporal signal modeling. We compare ICAD variants that use absolute timestamps (arrival and departure times) with those that rely on proxy signals (travel time, duration) for modeling visit temporal signals. Best (in bold) and second best performing models are highlighted.

Variant	Visit-level		Agent-level	
	
	AP	AUROC	AP	AUROC

ICAD_(Arrival+Duration)_	0.00067	0.7296	0.0025	0.5941
ICAD_(Arrival+Departure)_	0.0341	0.7265	0.0663	0.6853
ICAD_(Travel+Duration)_	0.0240	0.8108	0.0193	0.7685
ICAD_(Travel+Departure)_	**0.1610**	**0.8332**	**0.2561**	**0.9010**

**Table 6:** Effects of relative anomaly scores on NUMOSIM on both visit-level and agent-level scenario. Best (in bold) and second best performing models are highlighted.

Dataset	Level	Scoring Method	AP	AUROC

NUMOSIM	Visit-level	JLD	0.0109	0.8070
		NLL	0.1566	0.8165
		Bi-CDF	0.0795	0.8317
		Relative Scoring	**0.1610**	**0.8332**

	Agent-level	JLD	0.0253	0.8571
		NLL	0.2534	0.8924
		Bi-CDF	0.2397	0.8928
		Relative Scoring	**0.2561**	**0.9010**

## CCS Concepts

• Information systems → Location based services; • Computing methodologies → Anomaly detection.

## A Appendix

### A.1 Proof of 4.5.2

We begin by proving the following lemma:

#### Lemma A.1.

*Let* $p(x)=\Sigma_{k}\pi_{k}f_{k}(x)$ *be the probability density function (PDF) of a Gaussian Mixture Model (GMM) over a continuous random variable* x, *where each* $f_{k}(x)$ *is* k’*th Gaussian component with mean* $\mu_{k}$ *and prior mixture weight* $\pi_{k}$. *Then the marginal density evaluated at the component modes serve as an upper bound on the marginal density at any point* x, *i.e,*

(19)
$$\Sigma_{k}\pi_{k}f_{k}\left(x\right)\leq \Sigma_{k}\pi_{k}f_{k}\left(m_{k}\right).$$

#### Proof.

$f_{k}(x)$ is a gaussian distribution whose $\mu_{k}$ equals its mode $m_{k}$. By definition, $m_{k}$ is the point with highest density. Therefore, we have:

(20)
$$f_{k}(x)\leq f_{k}\left(m_{k}\right)\;\forall x$$

Multiplying both sides by $\pi_{k}$ preserves the inequality:

(21)
$$\pi_{k}f_{k}(x)\leq \pi_{k}f_{k}\left(m_{k}\right)\;\forall x$$

Summing over all components yields:

(22)
$$\Sigma_{k}\pi_{k}f_{k}(x)\leq \Sigma_{k}\pi_{k}f_{k}\left(m_{k}\right)\;\forall x$$

As a result, the relative log-likelihood between the mixture evaluated at the component modes and at any point x is always non-negative:

(23)
$$log\left(p\left(m_{k}\right)\right)-log(p(x)\geq 0$$

This result justifies the use of the log-likelihood at GMM component modes as a stable upper bound reference when computing non-negative continuous anomaly scores, as described in Section 4.5.2.

##### A.2 Bi-CDF Anomaly Scoring

Let f(x) denote the estimated probability density of a continuous component x (i.e. arrival or departure time). We define the cumulative density function:

(24)
$$F\left(x\right)=Pr\left(X\leq x\right)=\int_{-\infty }^{x}f\left(t\right)dt.$$

Because anomalous observations deviate significantly from typical behavior, they appear in the low-probability “tails” at either end of the distribution. Therefore, we compute both tail probabilities:

(25)
$$t_{\ell }\left(x\right)=F\left(x\right),\;t_{u}\left(x\right)=1-F\left(x\right),$$

and define the CDF-based anomaly score by scaling the more extreme tail probability:

(26)
$$S_{CDF}(x)=2\times min\{F(x),1-F(x)\}$$

This mapping yields $S_{CDF}(x)\in [1,2]$, with larger values indicating greater anomalousness.
